# Comparative Analysis of the Mechanical Properties and Biocompatibility between CAD/CAM and Conventional Polymers Applied in Prosthetic Dentistry

**DOI:** 10.3390/polym16070877

**Published:** 2024-03-22

**Authors:** Bozhana Chuchulska, Mariya Dimitrova, Angelina Vlahova, Ilian Hristov, Zlatina Tomova, Rada Kazakova

**Affiliations:** 1 Department of Prosthetic Dentistry, Faculty of Dental Medicine, Medical University of Plovdiv, 4000 Plovdiv, Bulgaria; angelina.vlahova@mu-plovdiv.bg (A.V.); ilian.hristov@mu-plovdiv.bg (I.H.); zlatina.tomova@mu-plovdiv.bg (Z.T.); rada.kazakova@mu-plovdiv.bg (R.K.); 2 CAD/CAM Center of Dental Medicine, Research Institute, Medical University of Plovdiv, 4000 Plovdiv, Bulgaria

**Keywords:** 3D printing, CAD/CAM, PEEK, thermoplastic, prosthodontics, polymers

## Abstract

Modern media often portray CAD/CAM technology as widely utilized in the fabrication of dental prosthetics. This study presents a comparative analysis of the mechanical properties and biocompatibility of CAD/CAM (Computer-Aided Design/Computer-Aided Manufacturing) polymers and conventional polymers commonly utilized in prosthetic dentistry. With the increasing adoption of CAD/CAM technology in dental laboratories and practices, understanding the differences in material properties is crucial for informed decision-making in prosthodontic treatment planning. Through a narrative review of the literature and empirical data, this study evaluates the mechanical strength, durability, esthetics, and biocompatibility of CAD/CAM polymers in comparison to traditional polymers. Furthermore, it examines the implications of these findings on the clinical outcomes and long-term success of prosthetic restorations. The results provide valuable insights into the advantages and limitations of CAD/CAM polymers, informing clinicians and researchers about their suitability for various dental prosthetic applications. This study underscores the considerable advantages of CAD/CAM polymers over conventional ones in terms of mechanical properties, biocompatibility, and esthetics for prosthetic dentistry. CAD/CAM technology offers improved mechanical strength and durability, potentially enhancing the long-term performance of dental prosthetics, while the biocompatibility of these polymers makes them suitable for a broad patient demographic, reducing the risk of adverse reactions. The practical implications of these findings for dental technicians and dentists are significant, as understanding these material differences enables tailored treatment planning to meet individual patient needs and preferences. Integration of CAD/CAM technology into dental practices can lead to more predictable outcomes and heightened patient satisfaction with prosthetic restorations.

## 1. Introduction

The advancement of science and technology significantly impacts the evolution of dentistry, leading to the continual refinement and enhancement of prosthetic material qualities [1]. The interplay between oral tissues and prosthetic materials is paramount in ensuring effective prosthetics, patient satisfaction, and oral health equilibrium, sustaining tissue integrity and functions [2,3]. The utilization of materials for dental prostheses elicits various adaptive responses within the human body, particularly evident with removable prosthetic structures, as the oral mucous membrane reacts to foreign bodies [4]. Optimal materials for removable prosthetics exhibit qualities conducive to tissue preservation and attenuated adaptive responses, characterized by biologically neutral thermoplastics. These materials, devoid of residual monomers and toxic or allergenic components, demonstrate high biocompatibility, elasticity, plasticity, precision in construction, and mechanical strength [5,6]. The impact of biomaterials on the human body, including their degradation in the oral cavity and potential for bacterial colonization, is essential. Biomaterials used in prosthetic dentistry, or any medical application must undergo thorough evaluation for their biocompatibility and long-term effects:

- Biocompatibility and Human Body Impact: Biomaterials intended for use in the oral cavity must be compatible with the surrounding tissues. This involves assessing how the material interacts with the body, whether it causes any adverse reactions, and if it integrates well with the surrounding tissues without causing inflammation or immune responses.
- Degradation in the Oral Cavity: The degradation behavior of biomaterials in the oral environment is crucial for their longevity and performance. Factors such as exposure to saliva, enzymes, pH variations, and mechanical stresses can influence the degradation process. Understanding how biomaterials degrade over time helps in predicting their lifespan and the potential risks associated with degradation by-products.
- Bacterial Colonization and Biofilm Formation: Biomaterials in the oral cavity can serve as substrates for bacterial colonization, leading to biofilm formation. Biofilms are communities of microorganisms encased within a matrix of extracellular polymeric substances. These biofilms can harbor pathogenic bacteria, contributing to various oral diseases such as dental caries, periodontal diseases, and implant-associated infections. Therefore, it is crucial to assess the propensity of biomaterials to support bacterial growth and biofilm formation.

Polymeric materials are integral to dentistry, offering diverse applications due to their unique surface properties, mechanical characteristics, biological attributes, simplified processing, and cost-effectiveness [7]. Polymethyl methacrylate (PMMA), polyurethane (PU), polyethylene (PE), polycarbonate (PC), polyetherether-ketone (PEEK), polyethylene glycol (PEG), polydimethylsiloxane (PDMS), polylactic acid (PLA), poly(e-caprolactone) (PCL), acrylonitrile butadiene styrene (ABS), and polypropylene (PP) are commonly utilized polymers in dental contexts [8,9].

Sorption and solubility are prominently featured in numerous ISO standards related to dental products such as filling materials, denture base polymers, and temporary crowns and bridges. These standards recognize the importance of assessing how these materials interact with their environment, particularly in terms of their ability to absorb fluids (sorption) and their tendency to dissolve or leach components into surrounding media (solubility). Understanding and controlling these properties are crucial for ensuring the performance, longevity, and biocompatibility of dental materials in clinical applications.

The mechanical properties most extensively investigated in dental polymers include flexural strength, impact strength, and hardness [10]. Flexural strength assesses a material’s resistance to fracture, offering insights into its behavior under static loads, with higher values of this property being clinically significant in reducing prosthetic base fractures. Moreover, subjecting a prosthetic base to the three-point flexion test, a commonly employed method, allows for the simulation of its capacity to endure intraoral functional forces [11]. Notably, the three-point bending test has been endorsed by ISO standards as the preferred bending assessment for polymers, with clinical acceptability and satisfaction typically observed for values not falling below 65 MPa (ISO 20795-1:2013-Dentistry—Base polymers—Part 1) [12].

While the mechanical properties of these polymers are inherent to their bulk material, their interaction with oral tissues largely depends on surface characteristics, warranting the use of polymer coatings to improve biocompatibility [13]. These polymers find applications across various dental disciplines, including direct restorative procedures, prosthodontics, orthodontics, and implantology. Significantly, synthetic polyetheretherketone (PEEK) has emerged as a promising candidate for implant materials [14]. Employing 3D printing technology enables the fabrication of intricately detailed custom facial prostheses from polymers with ease [15]. Additionally, polymers play a crucial role in creating scaffolds for bone regeneration and developing tissues resembling dentin and pulp (BIOFLOAT™ 96-well plate, faCellitate, Mannheim, Germany). They are also utilized in manufacturing membranes for guided tissue regeneration and as carriers for drug delivery in treating various oral and periodontal conditions [10].

Biocompatibility is a critical consideration in the selection of dental polymers utilized in prosthetic dentistry [16]. These materials must demonstrate compatibility with the oral environment, ensuring minimal adverse reactions when in direct contact with the surrounding tissues. Dental polymers undergo rigorous biocompatibility testing to assess their suitability for clinical use. These evaluations often include assessments such as cytotoxicity, genotoxicity, and irritation potential, among others, following established standards like the USP Class IV and ISO 10993 [17]. Additionally, the stability of these polymers within the oral cavity is crucial to preventing degradation and ensuring long-term performance. Ensuring optimal biocompatibility of dental polymers is paramount to the success and longevity of prosthetic restorations, promoting patient safety and well-being in dental practice [18,19].

This narrative review aims to offer a thorough comparative examination of the current advanced stage of conventional and CAD/CAM polymers in prosthodontics, specifically emphasizing the mechanical properties and biocompatibility of the polymeric materials, and to outline their potential advantages and drawbacks [20]. To achieve this, an exhaustive search was conducted across reputable databases, including PubMed, Web of Science, and EMBASE, gathering the literature published between 2004 and 2024.

## 2. Materials and Methods

The search scope included articles detailing the application of conventional, milled, and 3D-printed polymers in prosthetic dentistry. This was accomplished by employing a combination of diverse keywords, such as “dentistry” OR “digital dentistry”, AND “polymers”, AND “3D printing” OR “rapid prototyping” OR “CAD/CAM” OR “additive manufacturing” OR “Milled” OR “digital prosthodontics” OR “biocompatibility” OR “mechanical properties”. A total of 255 titles were acquired from the electronic databases, and upon applying exclusion criteria, 137 articles related to conventional and 3D printed technology for removable dentures were identified.

The inclusion criteria ensured that only complete texts written in English were included for analysis. Furthermore, articles had to be peer-reviewed and published in reputable journals to ensure relevance and reliability. Additionally, studies focusing on human subjects were prioritized. Any articles not meeting these criteria were excluded. Additionally, articles published up to the current date were considered. Besides the electronic search, a supplementary manual examination of pertinent citations and references was conducted to enhance the review’s comprehensiveness. Any additional relevant studies identified through this manual search process were included for analysis.

The review process comprised three stages: title review, abstract assessment, and final article selection for full-text analysis. Initially, articles retrieved from databases were individually evaluated by three reviewers, and any discrepancies in selection were deliberated until consensus was achieved. Upon unanimous agreement, articles not meeting the predetermined inclusion criteria were excluded. Subsequently, the abstracts of selected articles underwent independent evaluation by the same reviewers, with those chosen proceeding to full-text acquisition. Finally, in the third stage, the full texts of the acquired articles were comprehensively analyzed.

## 3. Recent Assessment of the Mechanical Properties of Conventional Polymers Employed in Prosthodontics

Acrylic resins designed for utilization in prosthodontics require specific attributes, including mechanical robustness, chemical stability, biocompatibility, and favorable aesthetic properties [21]. Numerous modifications have been implemented to enhance the physical characteristics, longevity, and processing techniques, and decrease the fabrication time of PMMA resin materials [5,9]. Poly (methyl methacrylate) (PMMA) finds widespread use in dentistry due to its convenient properties which facilitate its straightforward application. PMMA plastics are notably employed in the fabrication of partial and complete prostheses due to their satisfactory tensile strength ranging from 48 to 62 MPa and compressive strength of 75 MPa [11]. These strength properties are influenced by factors including the composition of the dental resin, degree of polymerization, technological protocol, water sorption, and subsequent storage and use of the dentures. Table 1 illustrates the mechanical properties of polymers commonly used in prosthetic dentistry:

Ideally, acrylic resins should possess high impact strength to mitigate the risk of breakage when the prosthesis is dropped [10]. Unmodified acrylic plastics tend to be more brittle, and the addition of plasticizers aims to enhance their strength properties. In terms of hardness and durability, acrylic resins exhibit relatively low hardness and are prone to scratching or deformation. Polyvinyl materials demonstrate superior wear resistance compared to heat-curing and self-curing alternatives [22].

Additionally, polymerization shrinkage, ranging from 0.3% to 0.8% in volume, results in inadequate marginal adaptation, necessitating further clinical interventions and additional processing steps, thereby increasing patient discomfort and inconvenience [23].

There are inherent limitations in the mechanical properties of polymethyl methacrylate (PMMA) that may impact its clinical performance adversely. PMMA typically exhibits low impact and tensile strength, necessitating reinforcement with filler materials. Current polymers utilized in dental applications often feature dimethacrylate structures, which, upon polymerization, form a network that contributes to the overall reinforcement of composites [24]. In prosthetic dentistry contexts, the addition of EGDMA (Ethylene Glycol Dimethacrylate) and TEGDMA (Triethylene Glycol Dimethacrylate) to the MMA (Methyl Methacrylate) monomer before polymerization is preferred over utilizing bis-GMA (Bisphenol A-glycidyl methacrylate) and UDMA (Urethane Dimethacrylate) as a composite material for tooth fillings. The formation of cross-links within the polymeric matrix, facilitated by the double vinyl bonds present in each molecule, results in a more durable material in terms of mechanical, thermal, and chemical stability over time. Nonetheless, various approaches have been employed to enhance PMMA acrylic resin and augment its physical and mechanical properties, including the incorporation of diverse types of fibers, metal oxides, nanoparticles, and carbon-based fillers [25]. Poly(methyl methacrylate) (PMMA) polymerization proceeds through chain polymerization employing a free-radical mechanism. Due to the limitation of applying high temperatures to initiate monomer radicals, the initiation system commonly consists of two compounds: benzoyl peroxide and *N*,*N*-dimethyl-p-toluidine. In commercial practice, PMMA powder containing benzoyl peroxide and liquid methyl methacrylate (MMA) with an amine are typically packaged separately. [26]. This self-polymerizing system, often termed “cold polymerization” due to the absence of heating and curing under ambient conditions, yields PMMA polymer upon mixing the two components in specific ratios for a brief period. The resulting paste can be easily manipulated and shaped by the operator within a few minutes before rapidly solidifying. Following adjustments for shape and occlusion, the restoration is prepared for temporary cementation and subsequent use [27,28].

Thermoplastic prosthetic materials present a promising alternative to conventional acrylics, with nylon polyamides emerging as the pioneering synthesized thermoplastics circa 1950. The initial documented utilization of these materials in dental practice, specifically for the fabrication of removable prostheses, was credited to Arpad and Tibor Nagi, who also established the Valplast company in 1959 [29]. Thermoplastic materials utilized in dentistry encompass composite substances or copolymers with thermoplastic attributes. These materials offer benefits such as monomer-free composition, absence of toxic or allergenic additives, high biocompatibility, shape retention, plasticity, precision in fabrication, color diversity, and expanded applications in various prosthetic scenarios, including partial and total prosthetics, immediate and post-resection prosthetics, prosthetics post-implantation, and aesthetic enhancement of prostheses [30]. As of present, the following categories of thermoplastic materials have been identified and utilized in the fabrication of removable dental prostheses due to their elastic and flexible properties:

1. Acetals
2. Polyamides
3. Acrylic polymers (free from residual monomer)
4. Polyolefins
5. Polyesters

These materials are characterized by their monomer-free, high-molecular-weight compositions. The term “thermoplastics” denotes their ability to transition from a solid to a liquid state under specific temperatures [31]. Whether natural or synthetic, these macromolecular compounds consist of large molecules, with molecular masses ranging from several thousand to several million [19]. They all exhibit thermoplastic properties resulting from diverse chemical compositions and structures, featuring linear or minimally branched chains that permit repeated softening upon heating and subsequent hardening upon cooling. This process mirrors the cyclic melting and crystallization observed in metals without undergoing chemical alterations [31].

The properties of these compounds are influenced by factors such as molecular mass, chemical structure, chain shape, and length. The elongated molecular structure of thermoplastic masses confers flexibility and high mechanical resilience. Linear macromolecules within these compounds contribute to their high density and mechanical strength [9] but also necessitate specialized techniques, such as injection molding, due to the labor-intensive processes involved [8].

**Figure 1:**
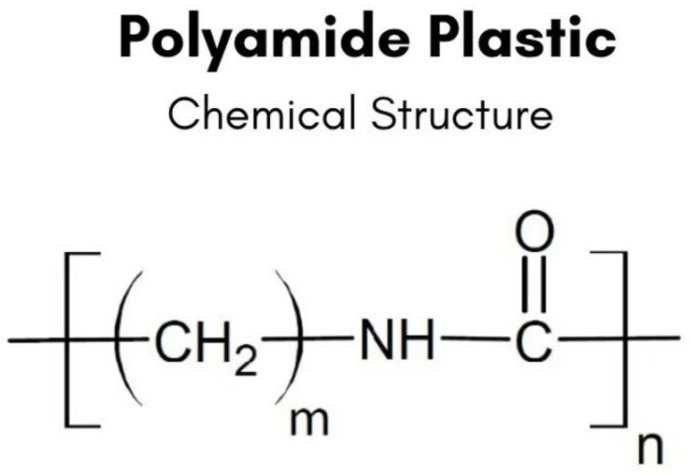
Chemical structure of polyamide.

Polyamides (Nylons) represent the most prevalent group of thermoplastic materials to date [3]. These heterochain compounds feature amide groups along the primary macromolecular chain, which are polar and capable of forming extensive hydrogen bonds with each other (Figure 1).

Two classes of polymers exist: homopolymers, derived from the polycondensation of diamine and a carboxylic acid, and copolymers, resulting from the copolymerization of multiple molecules of diamine and carboxylic acid. Among them are polyamides 6, 11, and 12, characterized by macromolecules composed of a single type of monomer, while polyamides 66, 69, and 610 incorporate two distinct monomer types [32].

**Figure 2:**
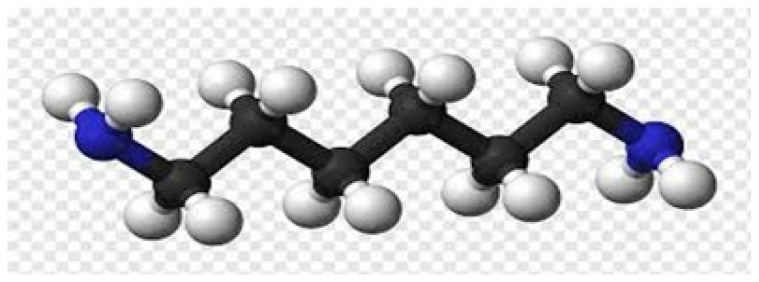
Spatial structure of polyamide.

In the solid state, the macromolecules of polyamides typically adopt a flat “zigzag” configuration (Figure 2). The presence of amide groups facilitates carbon bonds between the macromolecules, contributing to the high melting point of crystalline polyamides. These materials exhibit an amorphous, transparent, and glassy nature, with molecular weights ranging from 15,000 to 25,000 [12].

Polyamide exhibits insolubility in solvents such as alcohol, acetone, ketones, and various aromatic hydrocarbons. Its physical characteristics surpass those of certain metals, boasting resistance to mechanical abrasion, elevated temperatures, and hydrolyzing agents at room temperature, along with non-adsorption of liquids. In dental applications, non-toxic aliphatic polyamides are employed, characterized by solid-state macromolecules typically adopting a flat chain configuration comprising exceedingly lengthy chains containing upwards of 200,000 carbon atoms [33]. Aliphatic polyamides exhibit notable attributes, including high strength, wear resistance [1,2,10], decay resistance, and resilience to bacterial impact. Additionally, they find utility in medical contexts for fabricating artificial joints, blood vessels, valves, and similar components [18].

**Figure 3:**
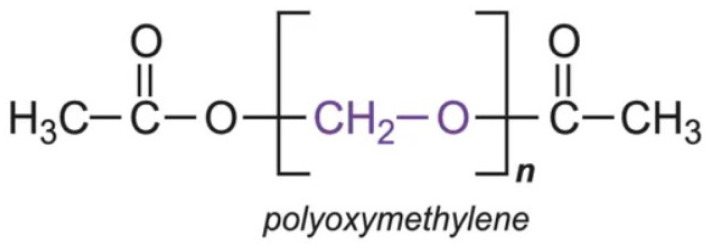
Chemical structure of polyoxymethylene.

Acetals, also known as polyoxymethylenes, were developed as a resilient plastic for dental use around 1970 [34]. This synthetic material is produced via gas-phase polymerization of paraformaldehyde (Figure 3).

Polyoxymethylene, comprised of a sequence of alternating methylene groups linked to oxygen atoms, possesses a crystalline structure devoid of residual monomers. It exists in both homopolymer and heteropolymer forms, with the latter exhibiting notably enhanced mechanical strength and stability [5]. As a solid material, it manifests itself as white, with a molecular weight ranging from 10,000 to 30,000. Although it does not boast high thermal and chemical stability, polyoxymethylene demonstrates exceptional hardness, a high working temperature, insolubility in chemical solvents, and remarkable wear resistance, facilitating its processing via injection molding [21]. Non-toxic and leaving no residue upon combustion, it showcases high resistance to dynamic loads, stability, strength, and hardness at temperatures near 100 °C, along with substantial wear resistance in frictional conditions. Furthermore, its hydrophobic nature prevents the retention of dental plaque due to its dense and smooth structure, ensuring color stability and odor retention [16].

The mechanical strength of dental materials surpasses that of acrylic plastics by up to 20 times. To mitigate potential toxic and allergic reactions in patients, chemical additives are eschewed in materials designed for dental applications. Prostheses crafted from polyoxymethylene rival those fabricated from metal in functionality, offering precise fitting, snug adherence to adjacent teeth and prosthetic sites, and dependable fixation [2,14].

**Figure 4:**
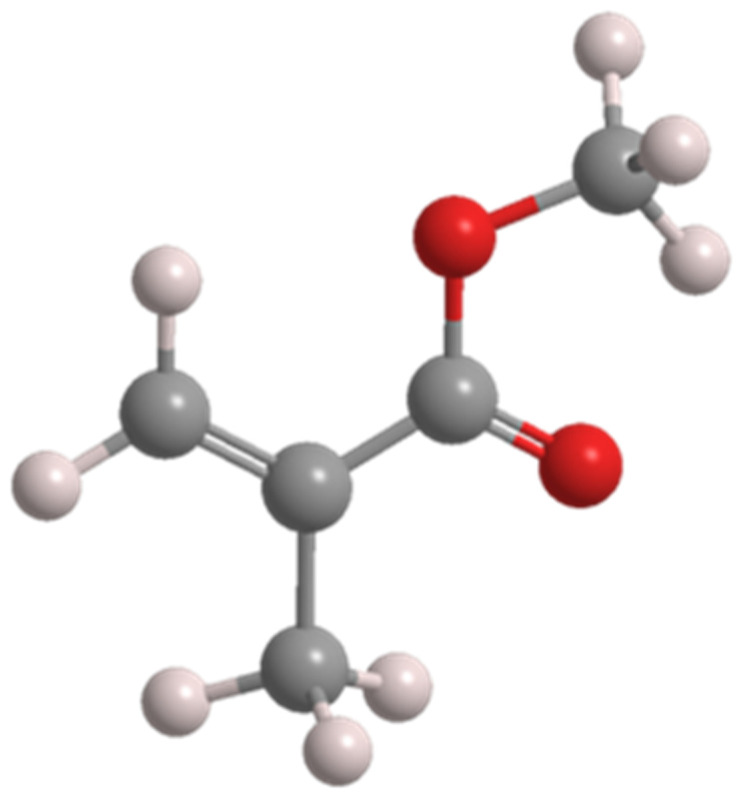
Spatial structure of acrylic resin (MMA).

Acrylic plastics devoid of monomers represent another category of thermoplastic materials. Chief among their characteristics is the absence of free monomers. Polymethyl methacrylate (-CH3–C[COOCH3] [CH3]–n-) constitutes an amorphous, transparent thermoplastic with a molecular weight reaching several million [35]. It dissolves in its own monomer and aromatic compounds, such as ketones and formic acid. Resistant to water, alcohol, simple aromatic hydrocarbons, saliva, and stomach acids, it poses no harm in biological environments while offering high wear resistance, mechanical strength, and favorable aesthetic attributes (Figure 4). 

When subjected to temperatures exceeding 120 °C, polymethyl methacrylate undergoes softening, transitioning into a highly plastic state amenable to molding. Visible depolymerization initiates at temperatures surpassing 200 °C, with the rate escalating significantly beyond 300 °C [11].

## 4. Biocompatibility of Traditional Polymers Applied in Prosthodontics

Materials described in the previous chapter are classified as biocompatible, rendering them suitable for fabricating prosthetic structures for patients sensitive to monomeric products. They exhibit resistance to weak acids, bases, and alcoholic products up to 30 °C. In such conditions, where the temperature exceeds the specified threshold of 30 °C, it is possible that the stability of these materials could be compromised. Higher temperatures can accelerate chemical reactions and potentially lead to degradation or changes in the properties of the materials. Therefore, if these materials are intended for use in environments where temperatures regularly exceed 30 °C, their performance and stability may be affected, and alternative materials with higher temperature resistance may be more suitable.

The presence of a smooth surface deters plaque accumulation, while their dense structure imparts resistance to loads generated during chewing, ensuring precise replication of the prosthetic field for a snug fit to the oral mucosa and improved adhesion and retention of complete dentures [11]. Following meticulous processing utilizing Computer-Aided Manufacturing (CAM) devices and subsequent polishing, they acquire a sleek surface that inhibits biofilm formation.

**Figure 5:**
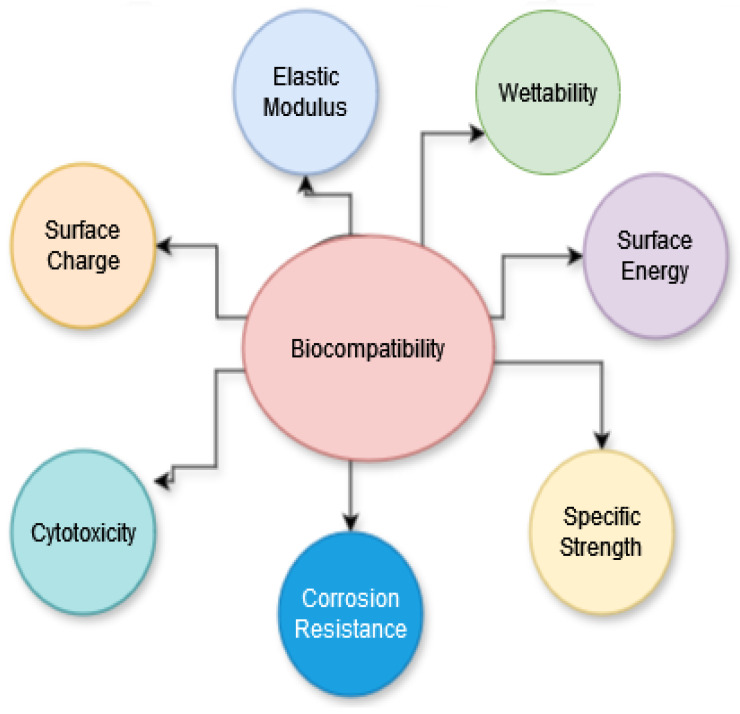
Factors affecting the biocompatibility of polymers applied in prosthodontics.

Figure 5 represents the factors affecting the biocompatibility of polymers applied in prosthodontics.

Polyolefins, encompassing thermoplastic materials such as polybutene, polyethylene, and polypropylene, are amorphous, transparent, tasteless, and odorless polymers with non-toxic properties. Their mechanical strength is contingent upon molecular weight, and they possess lower specific gravity than with a water lower density [36]. Recent years have seen their integration into dental practice owing to their favorable mechanical and aesthetic attributes. Predominantly semi-crystalline, they resist liquid absorption and maintain stable coloration, with polypropylene emerging as the most prevalent thermoplastic variant, boasting a molecular weight ranging from 75,000 to 200,000. Harmless and highly wear-resistant, polypropylene finds application in fabricating partial dentures [14].

Polyesters, comprising polymers featuring ester functional groups in their chains, encompass both natural (e.g., cutin) and synthetic (e.g., polycarbonate) varieties [37]. Exhibiting high impact resistance and resilience to external and atmospheric factors, they withstand temperatures ranging from −40 to +120 °C [7,10]. Flexible and biologically compatible, these thermoplastic materials are utilized for crafting temporary crowns, bridges, and, more recently, removable prostheses. Possessing commendable mechanical properties, they can withstand temperatures ranging from 230 to 290 °C, with a modulus of elasticity of approximately 1490 MPa. Transparent and easily polished, polyesters offer versatility in dental applications [38].

Polymeric dental structures situated within the oral cavity encounter a multitude of influences spanning the physical, chemical, and biological realms. These structures endure exposure to an aggressive chemical environment, including saliva, and endure substantial mechanical forces during mastication [39]. Conversely, the materials constituting these structures exert profound effects on the oral environment and overall systemic health. Thus, ensuring the longevity of materials and the preservation of their mechanical properties holds paramount significance in dental practice and patient satisfaction [12].

Despite the introduction of thermoplastic-based prosthetic materials as a notable advancement over conventional polymethyl methacrylate (PMMA), their utilization is recommended primarily in complex clinical scenarios, such as instances of patient allergies to acrylate monomers [39]. A recent research observed a considerable reduction in flexibility and bending capability post-application of thermoplastic dentures, even in comparison to PMMA, attributing the longer retention of mechanical properties to thermoplastics based on PMMA [11]. Studies indicate that exposure of these materials to fluctuating and humid environments leads to volume expansion due to water sorption, consequently diminishing their mechanical strength parameters, particularly in the case of polyamides [14]. Furthermore, polyamide-based thermoplastics exhibit decreased elasticity and flexibility, prompting recommendations for reinforcement with metallic elements [40]. Despite demonstrating lower water sorption compared to conventional acrylic plastics, polyamide thermoplastics exhibit reduced flexural strength and mechanical properties, as confirmed by recent investigations [10,16]. While possessing advantages such as reduced water sorption and solubility relative to PMMA, the diminished elasticity in some thermoplastic materials poses a risk of breakage and discomfort for users of thermal prostheses [18].

Certain thermoplastic prosthetic materials, designed as substitutes for metals, exhibit comparable tensile strength in simulated tests [41]. However, their higher elasticity and flexibility raise concerns regarding the potential overload of abutment teeth hooks. Investigations have highlighted positive attributes, including flexibility and modulus of elasticity, in monomer-free injectable PMMA-based materials, compared to conventional counterparts, facilitating easier mechanical processing and polishing, thereby enhancing surface quality and protection against bacterial contamination, and mitigating the development of subprosthetic stomatitis [20]. However, professional cleaning preparations and machining tools utilized during technological processing may inadvertently increase surface roughness and bacterial contamination risk [42]. The research underscores the interplay between prosthetic material surface structure and microbial colonization [25].

The primary pathogenic bacterium implicated in the development of subdenture stomatitis is Candida albicans [43]. This pathogen possesses the ability to colonize both the surface of prosthetic constructs and the mucosa beneath the prosthesis asymptomatically [27]. Conventionally, the propensity of acrylic plastics for bacterial colonization by pathogenic microorganisms has been attributed to their hydrophilicity, solubility, surface stress, and rough texture, which facilitates easier organism infiltration [23]. Injectable materials have been identified as beneficial for patients at high risk of subdenture stomatitis, with Aslanimehr et al. noting a significant reduction in bacterial adhesion to these materials [28]. They recommend meticulous mechanical surface treatment and thorough polishing of injection-molded prosthetic materials to minimize surface contamination [44].

While Sharabasy et al. determined bacterial adhesion to the surfaces of injection plastics to be unlikely [29], reports of subdenture stomatitis in patients utilizing polyamide prosthetic materials indicate engagement on both prosthetic surfaces and mucous membranes [45]. Polyamide injection materials exhibited maximum bacterial adhesion and colony formation, surpassing even conventional PMMAs [30]. Despite their susceptibility to surface defects and high roughness levels, these materials are challenging to polish, fostering pathogen colonization [34]. Modifying polymer surfaces appears to be the most promising approach for achieving antimicrobial effects and reducing bacterial attacks [46].

Despite certain drawbacks, injection materials remain a preferred alternative, likely due to the precision and efficiency of the injection molding process [38], which results in positive material attributes and low residual monomer levels, expanding their applications in dental practice.

## 5. Mechanical Properties and Biocompatibility of Contemporary Polymers, Used in Modern Prosthodontics

### 5.1. PEEK

Polyetheretherketone (PEEK) belongs to the group of polyariletherketones (PAEK), high-performance polymers that possess excellent mechanical properties, and environmental resistance and remain functionally unaffected at extreme conditions like high or low temperatures [39]. PEEK is a thermoplastic semicrystalline polymeric material with a glass transition temperature of around 143 °C and a melting temperature of around 340 °C. This material has been applied in medicine for orthopedic devices in spine surgery, orthopedic surgery, and maxillo-facial surgery for several decades because Young‘s elastic modulus (3–4 Gpa) and tensile strength (80–100 Mpa) of the material are close to that of the human bone which provides the long-term success of the treatment [40,41,42]. The mechanical properties of the material make it a significant candidate for dental use in cases where metal-free dental restorations are required [43,44,45]. In the field of dental medicine, PEEK can be used for the production of removable denture frameworks, fixed prosthetic restorations, splints, and implant abutments. To improve the process of osseointegration and bioactivity, modifications of the material are developed and nowadays PEEK dental implants are introduced to the market [46]. For veneered restorations, different pre-treatments of PEEK surfaces are proposed to achieve higher bond strength to the veneering materials [47]. To achieve the desired mechanical and biological properties modifications of the composition and the surface of the material are used [48,49]

Dental restorations can be produced from PEEK using either the conventional injection molding technique or CAD/CAM technologies which include subtractive and additive manufacturing methods. Milling (machining) is a subtractive process of removing material from a fabric-made disc of PEEK to create the desired shape of the final object designed in the CAD software 3Shape (3Shape Unite, Copenhagen, Denmark). PEEK can be also processed by additive manufacturing methods like FDM (fused deposition modeling) and FFF (fused filament fabrication) [50]. Limaye et al. found that 3D-printed PEEK has advantages over milled PEEK considering cell adhesion and biocompatibility, although it shows lower mechanical resistance [51]. By choosing appropriate printing parameters such as deposition direction, speed, printing temperature, and layer thickness it is possible to modify the mechanical properties of the final PEEK restoration [52,53]. 

The surface roughness of prosthetic restorations is of significant importance for bacterial adhesion and colonization of microorganisms. Mory et al. investigated the surface texture of high-performance PEEK materials and concluded that it is similar to that of Zirconia and that thermocycling did not cause adverse alterations [54]. Sanchez-Sobrado et al. also concluded that the aging conditions of the surrounding environment did not change the properties of PEEK [55]. The study of Wiessner et al. shows that if surface roughness may be eliminated as a factor determining microbial biofilm formation, then the type of material defines the level of bacterial colonization. They found the lowest biofilm accumulation on zirconia specimens, followed by titanium, and PEEK [56]. 

Polyetheretherketone can be used as a denture base material. According to the studies of Shrivastava et al., the flexural strength and hardness of PEEK are superior to those of heat-cured polymethyl methacrylate (PMMA) and make it suitable for frameworks of removable dentures [57]. Muhsin et al. also concluded that the mechanical properties of PEEK materials are better than those of PMMA [58]. Tushan et al. compared the impact strength and flexural strength of PEEK, CAD/CAM PMMA, and conventional heat-cured PMMA. They concluded that PEEK and CAD/CAM PMMA possess higher values of the tested characteristics and may be recommended as a better alternative to conventional PMMA for full denture production [59]. Wu et al. compared the retention and the fit of clasps for removable partial dentures made of PEEK and CoCr alloy and concluded that the PEEK clasp showed less fatigue deformation and could be applied for clinical use [60]. A combination of PEEK + PEEK in telescopic crown planning shows similar retention forces to other combinations of materials [61].

In cases when PEEK is used as the framework for fixed prosthetic restorations one of the major concerns that occur is the bond strength between PEEK and the veneering materials from one side, and the cementing agent from the other [62]. Sulfuric acid etching, alumina particle air abrasion, and chemical treatment of PEEK surface with monomer primers can significantly improve hydrophilic properties, surface free energy, and shear bond strength of the material [63,64,65]. Laser surface treatment of PEEK increases the shear bond strength to heat-cured PMMA and types of cement [66,67]. The clinical report after the six-month patient observation of Kimura et al. showed that PEEK crowns can be successfully used without the risk of abrasion of antagonists and change of occlusal forces [68]. In their study, Attia et al. concluded that although hot-pressed and CAD/CAM milled crowns had marginal and internal fit within the accepted clinical range, milled crowns had superior values of the parameters observed [69]. Nagi et al. found that in comparison with lithium disilicate restorations, PEEK endocrowns had a better internal fit and marginal gap [70]. Frameworks made of PEEK with Titanium bases had better vertical and passive fit than the Titanium frameworks [71]. Considering the stress distribution around implants, crowns made of PEEK have a similar effect to Zirconia ones [72]. Over-implant restorations with the PEEK framework provide less stress over the underlying bone due to the ability of the material to absorb the occlusal load [73]. According to another study, the PEEK bar provides a better fit of the mini abutments to the implants, even after mechanical cycling compared to the CoCr bar [74]. 

Another possible application of PEEK is for the production of post-and-core restorations and occlusal splints [75]. Glass fiber-reinforced PEEK provides excellent mechanical properties, shear bond strength, and biocompatibility when used for post-and-core restorations according to Zhao et al. [76]. 

PEEK can be successfully used in maxillo-facial surgery for repairing maxillofacial bone defects and production of obturators in combination with or as an alternative to other materials like PMMA [77,78,79,80]. 

In the field of implantology the modulus of elasticity, tensile strength, stable chemical properties, and wear resistance make PEEK a good alternative to Titanium implants [81]. In addition, PEEK is a biologically inert, hydrophobic material, which does not allow absorption of proteins and cell adhesion, thus negatively affecting the osseointegration process [82,83]. In orthopedic surgery attempts are made to decrease the level of bacterial biofilm formation by surface modification–after etching and removing the residual sulfuric acid, antimicrobial peptides (AMPs) can be fixed on the treated surface [84]. Different types of coatings may be used to improve osseointegration-bioactive ions, calcium phosphate, proteins, aforementioned peptides, and natural biopolymers like hyaluronic acid (HA) [85,86]. Synthetic hydroxyapatite (HA) may be used as a coating or as a reinforcing phase enhancing the bioactivity of PEEK [41,87,88,89]. According to Mishra et al. and other authors surface modifications of PEEK enhance biocompatibility, cell adhesion, cell proliferation, and osseointegration [90]. Yu et al. concluded that not only chemical composition but also surface morphology and implant architecture may influence bioactivity [91]. Studies by Mostafa et al. showed that surface treatment of PEEK with Nd: YAG laser combined with UV light or application of platelet-rich fibrin (PRF) may significantly improve biological properties and osseointegration process [92]. 

In dental implantology, PEEK may be utilized to produce both dental implants and implant abutments [93,94,95]. Fiber-reinforced PEEK composites used for dental implants and abutments even show stress distribution to the bone tissues [96]. Implant abutments made of PEEK show less accumulation of bacterial plaque on the surface compared to Titanium ones [97,98]. According to Ortega-Martínez et al., PEEK abutments may be used as a metal-free alternative to Titanium abutments for long-term interim restorations [99]. Based on the findings of Saravi et al., they can also be used as an alternative to Zirconia abutments [100]. PEEK implants induce less stress shielding than Titanium [101].

### 5.2. 3D-Printed Denture Base Resins (Additive Manufacturing)

In contrast to the traditional heat-cured PMMA typically used in making denture bases, 3D-printed resin relies on photo-polymerization. The duration of post-curing is crucial for optimizing material performance [102]. While initial curing occurs during printing through laser or light projection, final polymerization is achieved via additional curing in a light cure unit. A study found that photo-polymerized denture base material exhibited better mechanical properties than conventionally polymerized ones, although it did not assess any 3D printed denture base materials [94]. 

Manufacturers recommend different post-curing times (ranging from 20 to 60 min) based on various 3D printing techniques and photo-polymerized materials. Several researchers have explored the impact of post-curing times on 3D printed resins, confirming its significant influence on material properties [15,27,41]. However, research on the effects of post-curing time specifically on different denture base resins is lacking, warranting further investigation. To the best of the authors’ knowledge, no study has presented results on the combined effects of printing orientation and post-curing time using 3D-printed NextDent denture base resin [103].

Several authors explore the adjustability of mechanical properties and biocompatibility [13,104,105]. A study demonstrated the non-toxicity of urethane acrylate (UA)--based resins and their capability to modify mechanical properties [106,107]. Similarly, it was observed variations in mechanical strength and cytotoxicity, specifically noting the superiority of milled polymethylmethacrylate (PMMA) over 3D-printed resins in terms of mechanical strength. These studies underscore the significance of selecting materials to attain desired properties while ensuring safety. Additionally, Ujfalusi et al., in their examination of biocompatibility, expand beyond cytotoxicity to include inflammatory responses, offering a more comprehensive understanding of the biological effects of dental resins [108]. This research highlights the intricate nature of biocompatibility, encompassing a wider array of biological impacts beyond conventional measurements.

Heo et al. explored the biocompatibility of 3D-printed photopolymers, emphasizing the significance of material composition and its impact on biocompatibility [109]. The study’s zebrafish assay revealed varying levels of toxicity among materials, and notably, some materials showed reduced toxicity after ethanol treatment. This highlights the importance of both material composition and post-processing techniques as crucial factors in assessing the biological risks associated with 3D printing photopolymers. The study affirms the reliability of zebrafish assays as effective tools for quantifying toxicity in additive manufacturing (AM) materials [110].

Other studies investigated the mechanical strengths of dental resins [111]. Stansbury et al. [112] compared various resins used in splints, finding similar biocompatibility profiles except for one outlier, whereas other researchers [113] stressed the importance of post-processing washing steps in enhancing biocompatibility. Both studies underscored the critical aspect of selecting biocompatible materials, although the factors influencing this property varied between the two investigations [114].

Srinivasan et al. examined both biocompatibility and mechanical properties, similar to the aforementioned studies, but also included surface roughness in their analysis, providing a more comprehensive evaluation of material characteristics [115]. Their findings, indicating similar biocompatibility across different manufacturing methods, align with the consensus of other studies that many dental resins exhibit biocompatibility [63].

### 5.3. Milled Denture Base Resins (Subtractive Manufacturing)

The onset of the new century has seen the adoption of CAD/CAM technology for the fabrication of dentures [116]. These CAD/CAM materials not only offer pleasing aesthetics but also demonstrate durability [117]. Furthermore, their processing is efficient, fabrication is swift, and they provide accurate marginal fit [118]. The mechanical strength and clinical longevity of such prostheses are reliably predictable [119]. The mechanical properties of milled denture base resins are significantly influenced by their composition [120]. The properties such as hydrophilicity, mobility, and kinetic parameters are determined by the molecular structure of the co-monomers used. It has been observed that acrylic resins with lower degrees of conversion tend to demonstrate weaker mechanical characteristics [36]. The increased flexural strength observed in CAD-CAM specimens can be linked to their higher degrees of conversion. However, reduced SH (surface hardness) values may suggest a lower degree of conversion (Dc) of the discs [121]. Conversely, inferior mechanical properties observed with 3D printing techniques may stem from layering constructed parallel to the load direction, leading to weak adhesion between successive layers [122].

The reported findings regarding the flexural strength (FS) of milled denture base materials varied among studies [123]. Several authors found that heat-polymerized PMMA exhibited superiority over milled PMMA [11,26]. However, it was concluded by others that milled PMMA demonstrated better mechanical properties compared to heat-polymerized PMMA [30,117]. Perea-Lowery et al. [110] demonstrated significant variation in mechanical properties among the tested materials, with heat-polymerized PMMA showing the highest FS value, although the performance of all resins was deemed satisfactory [124,125]. These findings suggest the potential applicability of using heat-polymerized PMMA for denture fabrication, as CAD/CAM resins did not exhibit superior mechanical properties. Al-Dwairi et al. [126] previously demonstrated better FS, impact strength, and flexural modulus of milled PMMA compared to heat-polymerized resin. However, another study [121] failed to identify the source of residual monomer or report significant differences between different PMMA types.

Photopolymerization of solvent-free resins offers economic advantages and numerous applications in dentistry. For optimal clinical performance, these resins should possess high curing rates after the post-polymerization process, storage stability, low viscosity, and adequate biological properties, resulting in a final product with high mechanical and physical properties [127].

Another study [128] reported the highest FS with milled PMMA, followed by heat-polymerized PMMA, and lastly, 3D-printed PMMA. This study was the only one included in this review that evaluated the FS of 3D-printed PMMA. Although the 3D-printed material exhibited the lowest FS value, it met the ISO recommendation for FS (65 MPa) [129] and is suggested as an option for the fabrication of denture bases. The variation in reported values could be attributed to the material’s structure [130,131]. However, the downside of 3D-printed acrylic is its low double-bond conversion compared to other types of acrylic resins, which could potentially affect its mechanical properties. Due to the limited number of studies, additional research is recommended to draw conclusions regarding the mechanical properties of 3D-printed denture base PMMA and to evaluate its suitability for denture fabrication with properties comparable to those of milled or heat-polymerized PMMA [41,132].

Nevertheless, in addition to the mechanical properties related to biocompatibility, it must be considered that these materials are characterized by optical properties comparable to ceramic materials, and therefore, they increasingly play a decisive role in clinical practice [133].

According to the findings of Ferrini et al., the efficacy of a particular CAD/CAM system, concerning marginal adaptation, is contingent upon the type of restorative material used [134]. Marginal adaptation holds significant clinical importance, as a broad marginal gap can predispose to postoperative sensitivity, secondary decay, margin discoloration, unattractive aesthetics, and mechanical shortcomings. Various studies have reported different marginal gap measurements for CAD/CAM lithium disilicate, ranging from 33.30 μm to 84 μm [135,136]. Differences in CAD/CAM systems can significantly influence marginal fit, as evidenced by studies comparing zirconia, lithium disilicate, and composite crowns fabricated using different systems [137]. Factors such as the scanning process and milling machine axes can contribute to discrepancies in marginal adaptation. Overall, these studies indicated that the marginal adaptation measurements obtained for both CAD/CAM composite blocks and IPS E.max restorations fell within the acceptable clinical range.

## 6. Conclusions

The use of CAD/CAM techniques in prosthodontics may present a viable option for enhancing the mechanical properties of dental polymers, although their clinical efficacy requires additional trials and investigations. Despite the multitude of available polymerization methods and techniques, compression molding utilizing a water bath yields satisfactory mechanical outcomes. However, other innovative methods are still in the preliminary trial stages and therefore necessitate further laboratory assessment. Additionally, it is crucial to consider the biocompatibility of these materials, as any modifications or enhancements should not compromise their safety when used in clinical settings. Further research should also explore the biocompatibility aspect alongside mechanical evaluations to ensure the overall suitability of these materials for dental applications.

## Figures and Tables

**Table 1:** Mechanical properties of polymers used in prosthetic dentistry.

Polymer Type	Flexural Strength (MPa)	Elastic Modulus (GPa)	Toughness (MPa)	Hardness (Shore D)
Conventional PMMA	65–90	2–3	3–10	75–95
CAD/CAM (Milled) Polymers	80–120	2–5	5–15	70–85
Composite (Laboratory) Resins	80–100	2–4	5–12	65–80
Thermoplastic Polyurethane	25–45	0.5–1.5	15–25	70–90
Polyetheretherketone (PEEK)	90–120	3–4	5–15	80–90
Polylactic Acid (PLA)	50–70	3–4	5–15	65–75
Acrylonitrile Butadiene Styrene (ABS)	30–50	2–3	5–12	75–85
Stereolithography (SLA) Resins	60–80	2–4	5–12	70–85

## Data Availability

Data are contained within the article.

## Abbreviations

ABS	acrylonitrile butadiene styrene
AM	additive manufacturing
CAD/CAM	computer-aided design/computer-aided manufacturing
CD	complete dentures
EGDMA	Ethylene Glycol Dimethacrylate
ISO	International Organization of Standardization
PC	polycarbonate
PCL	poly[e-caprolactone]
PDMS	polydimethylsiloxane
PE	polyethylene
PEEK	polyetheretherketone
PEG	polyethylene glycol
PLA	polylactic acid
PMMA	polymethyl methacrylate
PP	polypropylene
PU	polyurethane
STL	stereolithography, standard triangle language, standard tessellation language
TEGDMA	Triethylene Glycol Di-methacrylate
3D	three dimensional
